# Tadalafil Promotes the Recovery of Peripheral Neuropathy in Type II Diabetic Mice

**DOI:** 10.1371/journal.pone.0159665

**Published:** 2016-07-20

**Authors:** Lei Wang, Michael Chopp, Alexandra Szalad, XueRong Lu, LongFei Jia, Mei Lu, Rui Lan Zhang, Zheng Gang Zhang

**Affiliations:** 1 Department of Neurology, Henry Ford Hospital, 2799 W. Grand Boulevard, Detroit, Michigan, 48202, United States of America; 2 Department of Physics, Oakland University, Rochester, Michigan, 48309, United States of America; University of Edinburgh, UNITED KINGDOM

## Abstract

We previously demonstrated that treatment of diabetic peripheral neuropathy with the short (4 hours) half-life phosphodiesterase 5 (PDE5) inhibitor, sildenafil, improved functional outcome in diabetic db/db mice. To further examine the effect of PDE5 inhibition on diabetic peripheral neuropathy, we investigated the effect of another potent PDE5 inhibitor, tadalafil, on diabetic peripheral neuropathy. Tadalafil is pharmacokinetically distinct from sildenafil and has a longer half-life (17+hours) than sildenafil. Diabetic mice (BKS.Cg-*m*+/+*Lepr*^*db*^*/J*, db/db) at age 20 weeks were treated with tadalafil every 48 hours for 8 consecutive weeks. Compared with diabetic mice treated with saline, tadalafil treatment significantly improved motor and sensory conduction velocities in the sciatic nerve and peripheral thermal sensitivity. Tadalafil treatment also markedly increased local blood flow and the density of FITC-dextran perfused vessels in the sciatic nerve concomitantly with increased intraepidermal nerve fiber density. Moreover, tadalafil reversed the diabetes-induced reductions of axon diameter and myelin thickness and reversed the diabetes-induced increased g-ratio in the sciatic nerve. Furthermore, tadalafil enhanced diabetes-reduced nerve growth factor (NGF) and platelet-derived growth factor-C (PDGF-C) protein levels in diabetic sciatic nerve tissue. The present study demonstrates that tadalafil increases regional blood flow in the sciatic nerve tissue, which may contribute to the improvement of peripheral nerve function and the amelioration of diabetic peripheral neuropathy.

## Introduction

Phosphodiesterase-5 (PDE5) is highly specific for hydrolysis of cyclic nucleotides monophosphate (cGMP). PDE5 inhibitors are primarily used as pharmacological agents for the treatment of erectile dysfunction (ED) and pulmonary hypertension [1–3]. Peripheral neuropathy is a chronic complication of diabetes. Human and experimental studies have demonstrated that the PDE5 inhibitor, sildenafil, reduces symptoms of peripheral neuropathy [4, 5]. Our previous studies showed that hyperglycemia upregulated PDE5 expression and suppression of PDE5 by sildenafil increased cGMP levels and significantly improved neurovascular function and neurological outcome in diabetic mice, indicating that sildenafil has a beneficial effect on the treatment of diabetic peripheral neuropathy [6, 7]. To verify that the effect of sildenafil on diabetic peripheral neuropathy is not specific to sildenafil, we employ another PDE5 inhibitor, tadalafil, which is structurally and pharmacokinetically distinct from sildenafil. Tadalafil has a half–life of over 17 hours and its effect can last for up to 36 hours, while sildenafil has a half-life of 4 hours [8].

Diabetic peripheral neuropathy is a chronic disease and a short acting treatment may not be an optimal therapeutic approach. The considerably longer duration of action for tadalafil may permit less frequent dosing, and could potentially reduce adverse effects associated with treatment. For example, the efficacy of sildenafil is affected by certain foods [9]. However, the absorption and activity of tadalafil is unaffected by food ingestion, age, diabetes, or mild to moderate hepatic insufficiency [8, 10]. Tadalafil is safe and effective for the treatment of ED. Moreover, tadalafil is less expensive than sildenafil. Therefore, studying the effect of tadalafil on diabetic peripheral neuropathy is warranted as a potential treatment.

Tadalafil increases blood flow in ischemic brain and improves neurological outcome in ischemic rats [11]. Patients with ED treated with tadalafil have reduced diabetic peripheral neuropathy symptoms [12]. However, the role of tadalafil in diabetic neuropathy has not been fully investigated.

In this study, we investigated whether tadalafil is effective and safe for the treatment of diabetic neuropathy.

## Materials and Methods

### Ethics statement

All experimental procedures were carried out in accordance with NIH Guidelines for the Care and Use of Laboratory Animals and were approved by the Institutional Animal Care and Use Committee of Henry Ford Hospital (IACUC#1398). Male BKS. Cg-*m*+/+*Lepr*^*db*^*/J* (db/db) mice (Jackson Laboratories, Bar Harbor, Maine) aged 20 weeks were used. Age-matched heterozygote mice (db/m), a non-penetrant genotype (Jackson Laboratories), were used as the control animals.

### Tadalafil treatment

db/db mice at the age of 20 weeks were treated with tadalafil at a dose of 10 mg/kg (orally administered, p.o. Lilly USA, LLC.), every other day for 8 weeks (n = 15/group). db/db mice (n = 15/group) at the same age treated with the same volume of saline were used as a control group. Age-matched non-diabetic db/m mice treated with the same volume of saline (n = 15/group) were used as an additional control group. All mice were sacrificed 8 weeks after treatment. Dose and frequency of tadalafil were selected based on our published studies [11].

Levels of blood glucose, triglyceride, and A1C were measured using an instant check meter (Roche Diagnostics, Indianapolis, IN), CardioChek PA Analyzer and Triglyceride Test Strips (Polymer 285 Technology system), and A1C Now+ MULTI-TEST A1C SYSTEM, respectively, according to the manufacturer’s instructions. Blood glucose levels, body weight and functional tests were measured before the treatment as a baseline and then every 2 weeks until sacrifice. Triglyceride and A1C levels were measured prior to the treatment and at the end of the experiment (8 weeks after the initial treatment). Electrophysiological measurements were performed before the treatment and then every 4 weeks until sacrifice. All procedures and analyses were performed by investigators who were blinded to the treatment administered.

### Neurophysiological measurements

Sciatic nerve conduction velocity was assessed with orthodromic recording techniques, as previously described [6, 13]. Briefly, mice were anesthetized with ketamine/xylazine (i.p., 100/10 mg/kg). The stimulating electrodes were plated at the knee and sciatic notch. Trigger single square wave current pulses were delivered using an isolated pulse stimulator (Model 2100, A-M Systems, Everett, WA). The simultaneous electromyographys were recorded by two sterilized electrodes placed in the dorsum of the foot with a Grass Amplifier (Model P5, Grass Instruments, Quincy, MA). During the measurements, animal rectal temperature was maintained at 37 ± 1.0°C using a feedback controlled water bath. Motor nerve conduction velocity (MCV) and sensory nerve conduction velocity (SCV) were calculated according to a published study [13].

### Measurement of thermal sensitivity

To examine the sensitivity to noxious heat, plantar and tail flick tests were measured using a thermal stimulation meter (IITC model 336 TG combination tail-flick and paw algesia meter; IITC Life Science) according to published methods [14]. Briefly, mice were placed within a plexiglass chamber on a transparent glass surface and allowed to acclimate for at least 20 min. For plantar test, the meter was activated after placing the stimulator directly beneath the plantar surface of the hind paw. The paw-withdrawal latency in response to the radiant heat (15% intensity, cut-off time 30 sec) was recorded. For tail-flick test, the meter was set at 40% heating intensity with a cut-off at 10 sec. For both tests, at least five readings per animal were taken at 15 min intervals, and the average was calculated [15].

### Measurement of regional blood flow by laser Doppler flowmetry

Regional blood flow in the sciatic nerve was measured at the end of the experiments (8 weeks after the treatment) using laser Doppler flowmetry (LDF PeriFlux PF4, Perimed AB, Järfälla, Sweden) [15, 16]. Briefly, under anesthesia (ketamine/xylazine, i.p., 100/10 mg/kg, JHP Pharmaceuticals LLC. MI; LLOYD Inc. Lowa), the mouse was mounted on a Kopf stereotaxic apparatus. The left sciatic nerve was exposed in the mid-thigh region and animal rectal temperature was maintained at 37 ± 1.0°C during the measurement period using a feedback controlled water bath. Using a micromanipulator, a LDF probe was placed at the surface of the sciatic nerve and relative flow values expressed as perfusion units were recorded every 5 minutes for a total of 5 records. Regional blood flow values from non-diabetic mice were used as baseline values and data are presented as a percentage of baseline values.

### Staining myelin sheaths

The sciatic nerves were fixed in the 2.5% glutaraldehyde and 0.5% sucrose (Sigma) on PBS buffer for 6–8 hours, and then immersed in 2% osmium tetroxide (Sigma) for 2 hours. The specimens were then dehydrated with numerous alcohol passages and embedded in paraffin [17]. Semi-thin transverse sections (2-μm thick) were cut and stained with 1% toluidine blue and three semi-thin sections per mouse were analyzed. This method has been demonstrated as a reliable way to measure myelin sheaths [7, 18].

### Immunohistochemistry

The sciatic nerve tissues were fixed in 4% paraformaldehyde for immunohistochemistry and then embedded in paraffin according to published protocol [6]. Three cross sections (6-μm-thick) or three longitudinal sections (6-μm-thick) at 60 μm apart per animal were used [6].

Footpads were fixed in Zamboni's fixative for 2 hours, washed in PBS and then kept in 30% sucrose/PBS overnight at 4°C. The samples were embedded in OCT compound and stored at −80°C. Three longitudinal 20-μm-thick footpad sections from each mouse were prepared.

The following primary antibodies were used: polyclonal rabbit anti-NGF (1:500; Abcam, Cambridge, MA), polyclonal goat anti-PDGF (1:500; Santa Cruz Biotechnology, INC, Texas), monoclonal mouse anti-CD31 antibody (1:500, BD Biosciences, San Jose, CA), polyclonal rabbit anti-S100 (1:400, Abcam) and polyclonal rabbit anti-protein gene product 9.5 (PGP9.5, 1:1,000; MILLIPORE). Rabbit or goat IgG was used as a negative control. Sections were counterstained with 4*′*,6*-*Diamidino*-*2*-*phenylindole *(*DAPI*)* (1:5000, Thermo Scientific, Rockford, IL).

### Image acquisition and quantification

To examine microvascular perfusion in the sciatic nerve, fluorescein isothiocyanate (FITC)-dextran (2x10^6^ molecular weight, Sigma; 0.2 mL of 50 mg/mL) was administered intravenously to the mice 10 min before sacrifice [15, 19]. The sciatic nerve tissue was rapidly removed and placed in 2% of paraformaldehyde for 2 hours. The nerve tissue was whole-mounted and imaged under a 10x objective using a laser-scanning confocal microscope (Zeiss LSM 510 NLO, Carl Zeiss, Germany) [15, 19].

Thereafter, the nerves were embedded in OCT compound and cross cryosections (20 μm thick) prepared. Three sections at 60 μm intervals from each mouse were used for further image analysis. The cross sections were imaged under a 20x microscope objective (Carl Zeiss, Inc.) of a fluorescent microscope that was equipped with a MicroComputer Imaging Device (MCID, *Imaging Research Inc*.) [20]. Using the MCID system, the total number of FITC-dextran perfused vessels was counted and divided by the total tissue-area to determine vascular density [7].

For analysis of CD31 immunoreactive vascular perimeters, three cross sections spaced at 60 μm intervals from each mouse were used. Three fields of the view per section were randomly imaged under a 40x objective. CD31 immunoreactive vascular perimeters were measured using the MCID system [15].

For morphometric analysis of sciatic nerves, three sections spaced at 60 μm interval for each staining were used for analysis from each mouse. Three fields of view per section were randomly imaged under a 100x oil immersion objective (BX40; Olympus Optical Co. Ltd) via the MCID system. Myelinated fiber diameter, axon diameter, and myelin sheath thickness were measured. The g ratio (the quotient axon diameter/fiber diameter) was calculated to measure the degree of myelination. At least 200 myelinated fibers were measured per animal [6, 21].

Intraepidermal nerve fiber profiles were imaged under a 40x objective (Carl Zeiss,Inc.) via the MCID system. The number of nerve fibers crossing the dermal-epidermal junction were counted and the density of nerves is expressed as fibers/mm length of section [7]. Representative images of intraepidermal nerve fibers were obtained using a laser-scanning confocal microscope (Zeiss LSM 510 NLO, Carl Zeiss, Germany).

All analysis was conducted by an examiner who was blinded to the identity of the samples being studied.

### Statistical analysis

For functional tests, data were evaluated for normality. Ranked data or nonparametric approach will be considered if the data are not normally distributed. The repeated measure analysis of variance (ANOVA) was considered with dependent factor of time and independent factor of groups. The analysis started testing for group by time interaction, followed by the testing the main effect of group and subgroup analyses. Two-sample t-test or analysis of variance (ANOVA) was used to study the group difference on LDF, immunostaining, biochemistry and Western blot, respectively. The data are presented as mean ± SE. A value of p<0.05 was taken as significant.

## Results

### Tadalafil improves neurological function in the diabetic mouse

**Fig 1:**
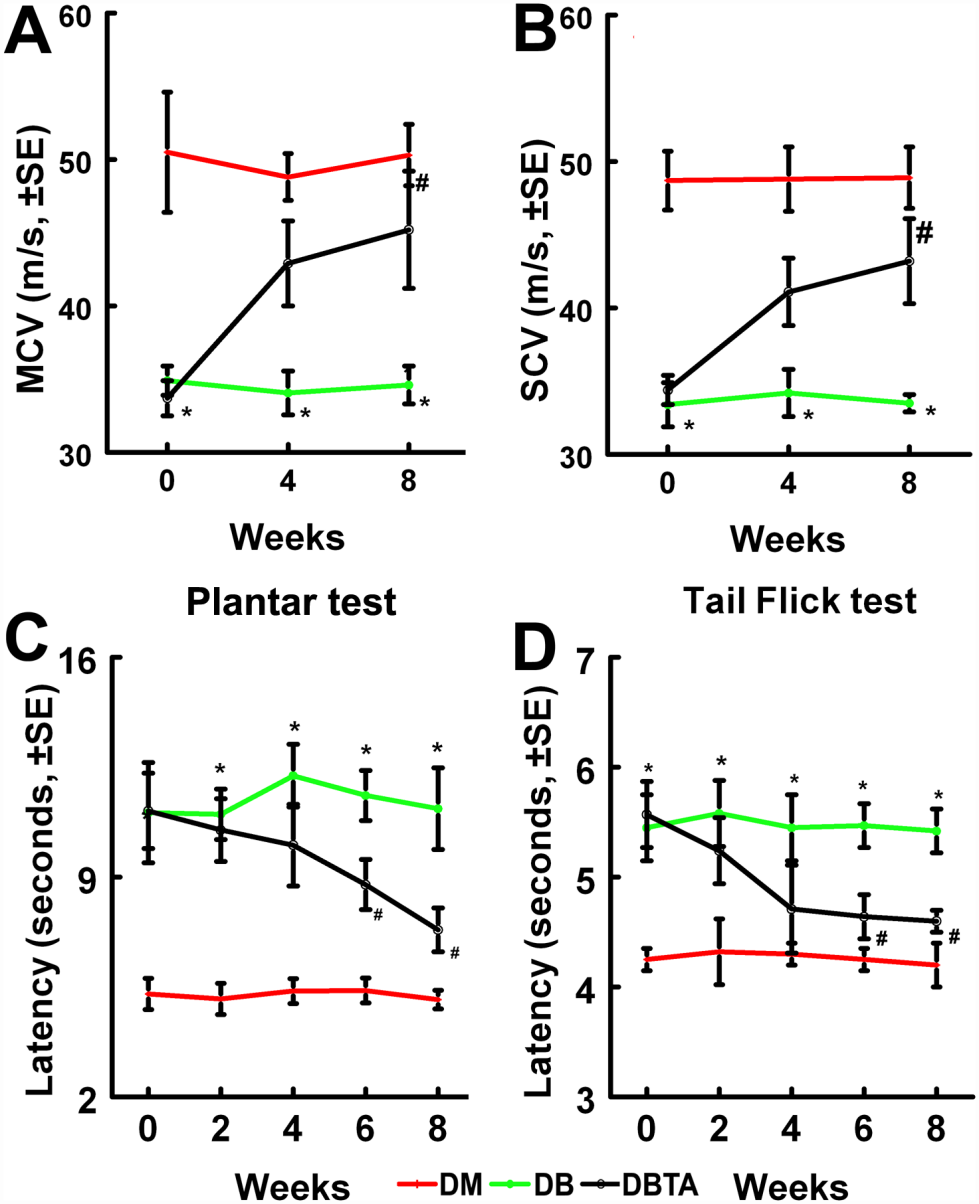
Tadalafil improves neurological function in the diabetic db/db mouse. Treatment of diabetic mice with tadalafil improves neurological function measured by MCV (A), SCV (B), Plantar test (C) and Tail flick test (D). *p<0.05 and #p<0.05 versus the non-diabetic mouse (DM) and the diabetic mouse treated with saline (DB), respectively. n = 10/group. Non-diabetic mouse = DM; diabetic mouse = DB; diabetic mouse treated with tadalafil = DBTA.

To examine the effect of tadalafil on diabetic peripheral neuropathy, diabetic db/db mice at aged 20 weeks, which exhibited severe peripheral nerve neurological deficits, were orally (p.o.) administered with tadalafil at a dose of 10 mg/kg every 48 hours for 8 consecutive weeks. Tadalafil treatment significantly improved diabetic-reduced motor and sensory conduction velocity (MCV and SCV) in the sciatic nerve measured by electrophysiological tests, while significant improvements of sensory function as measured by Plantar and Tail-Flick tests were evident starting at 6 weeks after the initial treatment compared with saline–treated diabetic mice (Fig 1). However, treatment with tadalafil did not significantly alter animal body weight (Table 1), blood glucose levels (Table 2), A1C and triglyceride levels (Table 3) compared to the saline treatment.

**Table 1:** Effect of tadalafil on body weight.

Body Weight (g)			
Groups	0 w	4 w	8 w
**DM-saline**	**32.9±0.6**	**34.3±0.7**	**34.1±0.8**
**DB-saline**	49.4±0.9*	46.1±1.1*	45.6±1.5*
**DBTA**	49.1±1.5*	46.3±1.8*	46.2±1.6*

Values are mean±SE.

*p<0.01 versus DM-saline group.

n = 10/group.

w = week, 0 w represents before the treatment, while other numbers indicate after the treatment. DM = non-diabetic mouse; DB = diabetic mouse; DBTA = diabetic mouse treatment with tadalafil.

**Table 2:** Effect of tadalafil on blood glucose.

Blood glucose (g/dl)			
Groups	0 w	4 w	8 w
**DM-saline**	**138.2±6.2**	**115.1±6.0**	**125.9±6.2**
**DB-saline**	562.2±9.4*	549.8±19.5*	565.0±8.0*
**DBTA**	574.1±16.4*	551.5±10.1*	578.3±12.1*

Values are mean±SE.

*p<0.01 versus DM-saline group.

n = 10/group.

w = week, 0 w represents before the treatment, while other numbers indicate after the treatment. DM = non-diabetic mouse; DB = diabetic mouse; DBTA = diabetic mouse treatment with tadalafil.

**Table 3:** Effect of tadalafil on A1C and Triglyceride.

	A1C		Triglyceride	
Groups	0 w	8 w	0 w	8 w
**DM-saline**	**4.6±0.1**	**4.5±0.1**	**55.6±2.1**	**70.4±13.2**
**DB-saline**	11.5±0.2*	11.4±0.7*	118±8.8*	137.2±5.3*
**DBTA**	11.8±0.3*	11.6±0.5*	98.4±9.8*	124.4±11.9*

Values are mean±SE.

*p<0.01 versus DM-saline group.

n = 10/group.

w = week, 0 w represents before the treatment, while other numbers indicate after the treatment. DM = non-diabetic mouse; DB = diabetic mouse; DBTA = diabetic mouse treatment with tadalafil.

### Tadalafil improves neurovascular function in the sciatic nerve

**Fig 2:**
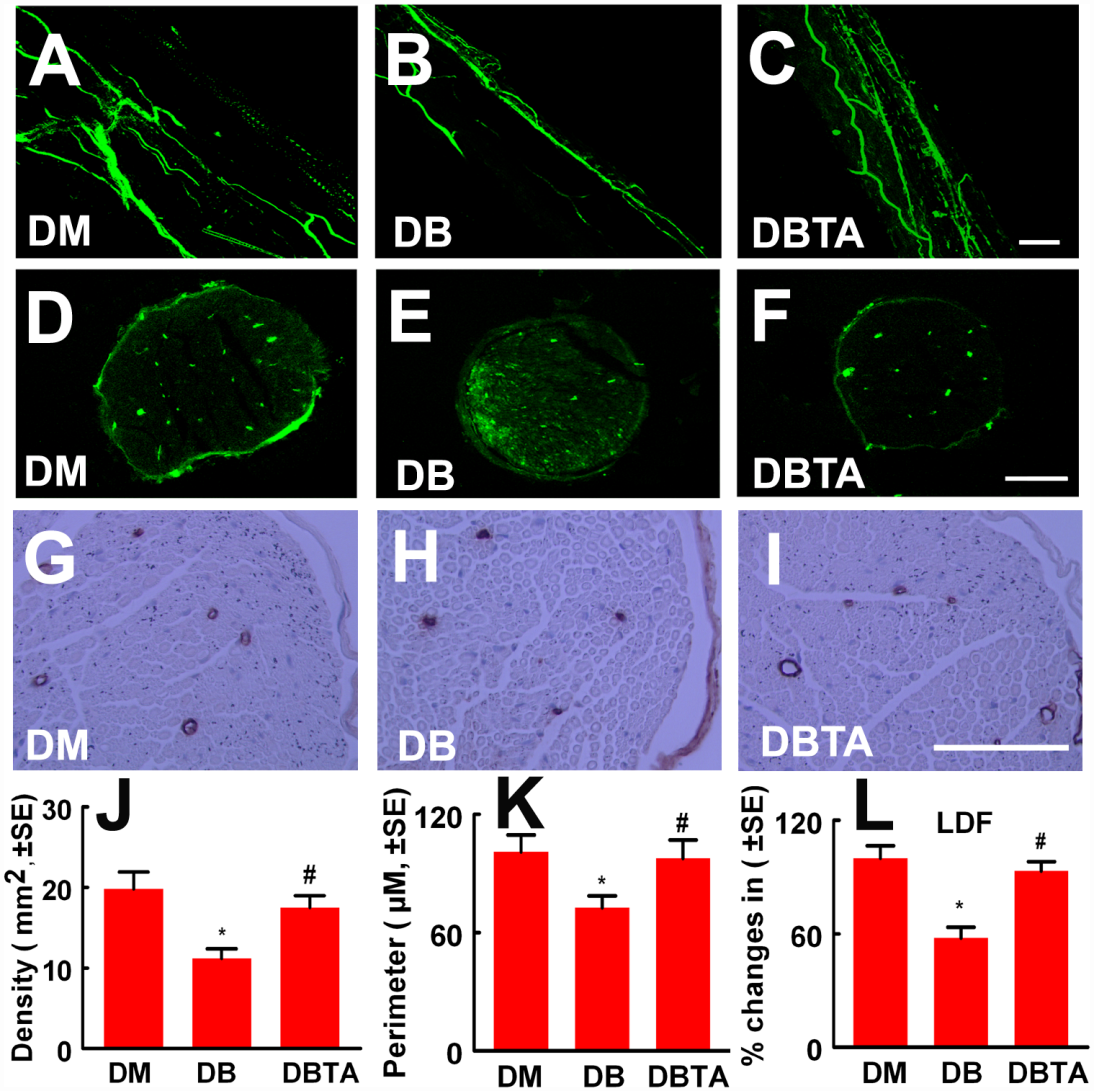
Tadalafil improves vascular function in the sciatic nerve tissue. Panels A to I show FITC-dextran perfused vessels from whole mounted (A to C) and cross sections (D to F) of the sciatic nerve tissue, and CD31 immunoreactive blood vessels at the cross section (G and I) of the sciatic nerve tissue from a representative non-diabetic mouse treated with saline (DM, A, D and G), diabetic mouse treated with saline (DB, B, E and H), and diabetic mouse treated with tadalafil (DBTA, 10 mg/kg, C, F and I). Panels J to L show quantitative data of density of FITC-dextran perfused vessels in cross section (J, n = 6/group), CD31 immunoreactive vascular perimeters (K, n = 6/group), and percentage changes of sciatic nerve blood flow with a reference of non-diabetic mouse at 100% (L, n = 5/group). *p<0.05 and #p<0.05 versus the non-diabetic mouse (DM) and the diabetic mouse treated with saline (DB), respectively. Bar = 100μm. DBTA = diabetic mouse treated with tadalafil.

Neurovascular dysfunction is a major cause of diabetic peripheral neuropathy [22, 23]. Using a laser Doppler flowmetry (LDF) system, longitudinal measurements of regional blood flow at the sciatic nerve revealed that tadalafil treatment significantly improved blood flow in the diabetic sciatic nerve (Fig 2). In parallel with blood flow results, confocal images acquired from whole-mount of the sciatic nerve tissue showed that diabetes induced substantial reduction of FITC-perfused blood vessels compared to that in non-diabetic mice, whereas tadalafil treatment markedly increased the number of FITC-dextran perfused vessels in diabetic sciatic nerve tissue (Fig 2). In addition, quantitative analysis of FITC-perfused blood vessels and CD31 immunoreactive vessels in cross sections of the sciatic nerve tissue revealed that tadalafil treatment markedly increased microvascular density and vascular perimeters in the sciatic nerve tissue compared to the diabetic mice treated with saline, respectively (Fig 2). Collectively, these data indicate that tadalafil improves vascular perfusion in the sciatic nerves tissue of diabetic mice.

The increase of vascular perfusion is associated with axonal regeneration in the diabetic patient [24]. To examine whether an increased vascular perfusion is associated with alteration of the peripheral nerve, morphometric changes of the sciatic nerves and intraepidermal nerve fibers were analyzed.

**Fig 3:**
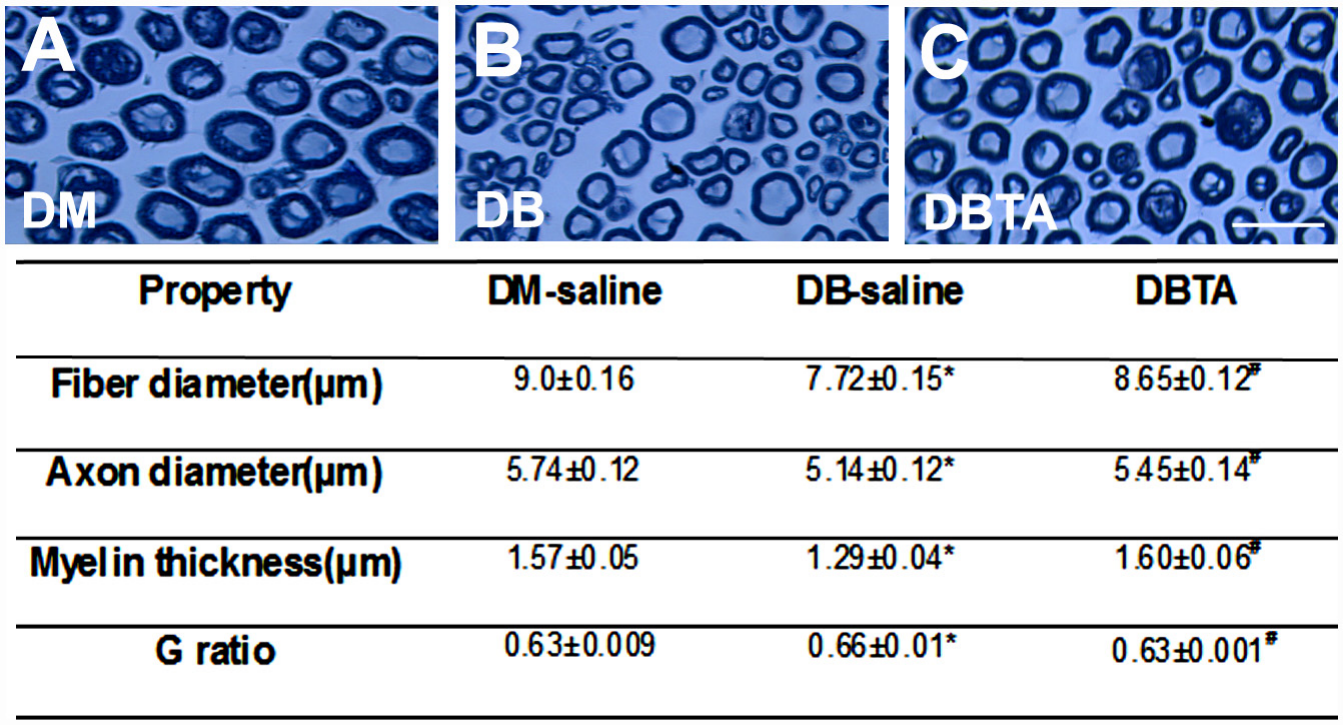
Tadalafil increases axonal remodeling in diabetic db/db mice. Panels A to C show semi-thin toluidine blue-stained cross sections of sciatic nerves from a representative non-diabetic mouse (DM, A), diabetic mouse treated with saline (DB, B), and diabetic mouse treated with tadalafil (DBTA, C). The table shows the effect of tadalafil on histomorphometric parameter of sciatic nerves. Values are mean±SE. *p<0.05 and #p<0.05 versus the non-diabetic mouse (DM) and the diabetic mouse treated with saline (DB), respectively. n = 10/group. Bar = 20μm. DBTA = diabetic mouse treated with tadalafil.

**Fig 4:**
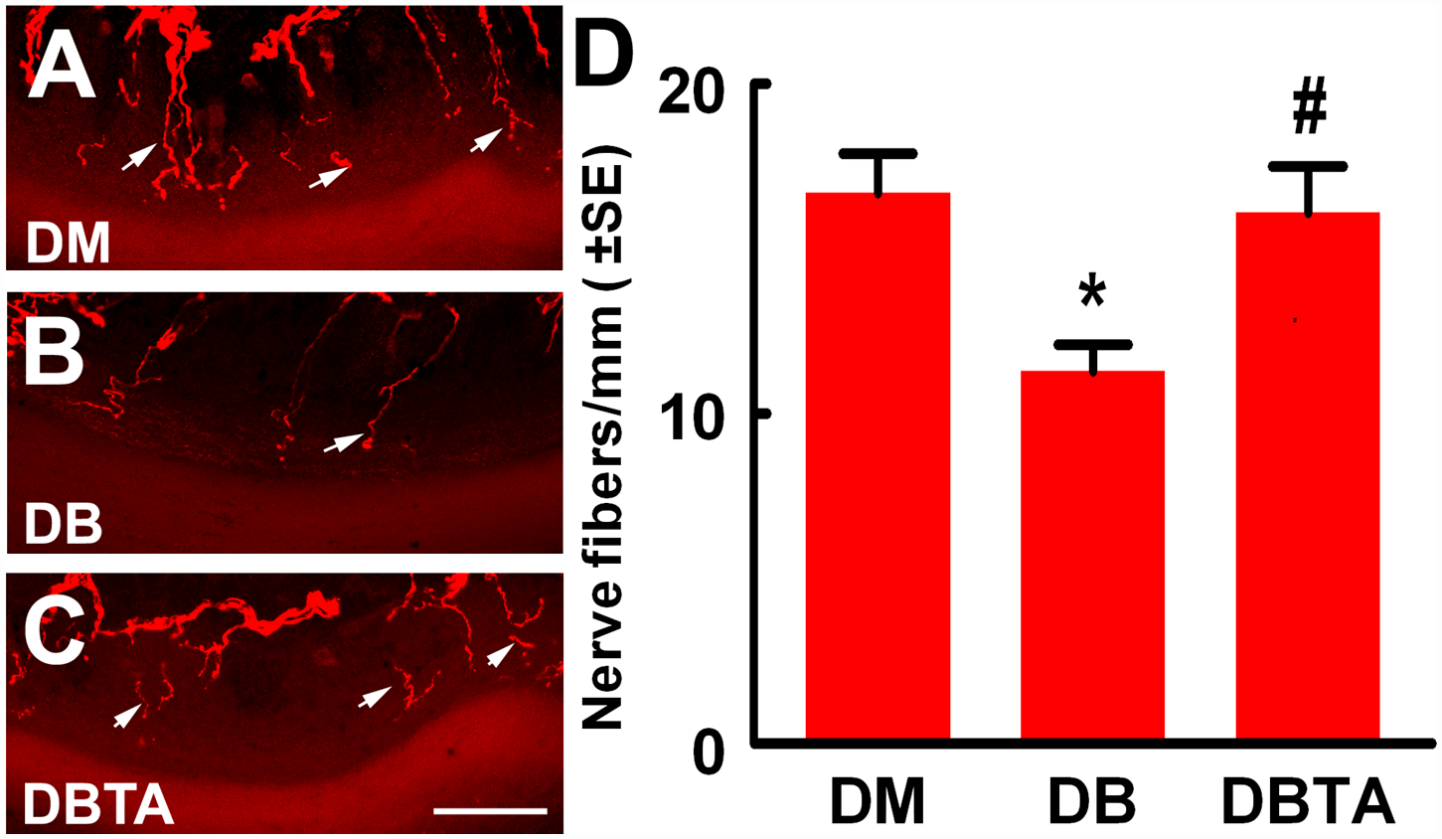
Tadalafil increases intraepidermal nerve fiber density in diabetic db/db mice. Panels A to C show PGP 9.5 immunoreactive intraepidermal nerve fibers (red, arrows) in the plantar skin from a representative non-diabetic mouse treated with saline (DM, A), diabetic mouse treated with saline (DB, B) and diabetic mouse treated with tadalafil (DBTA, C). Panel D shows quantitative data. *p<0.05 and #p<0.05 versus the non-diabetic mouse (DM) and the diabetic mouse treated with saline (DB), respectively. n = 10/group. Bar = 50μm. DBTA = diabetic mouse treated with tadalafil.

Histopathological analysis of sciatic nerve on semi-thin coronal sections showed that diabetic mice treated with tadalafil exhibited increased sciatic nerve fiber diameters and myelin sheath thickness, and decreased the g-ratio (axon diameter/fiber diameter) (Fig 3). Compared to diabetic mice treated with saline, diabetic mice treated with tadalafil also revealed a marked increase of PGP 9.5 positive intraepidermal nerve fiber density in the plantar skin tissue (Fig 4). Together, these data suggest that tadalafil-improved vascular perfusion is associated with enhancement of axonal remodeling in sciatic nerves.

### Tadalafil treatment increases NGF and PDGF-C proteins

**Fig 5:**
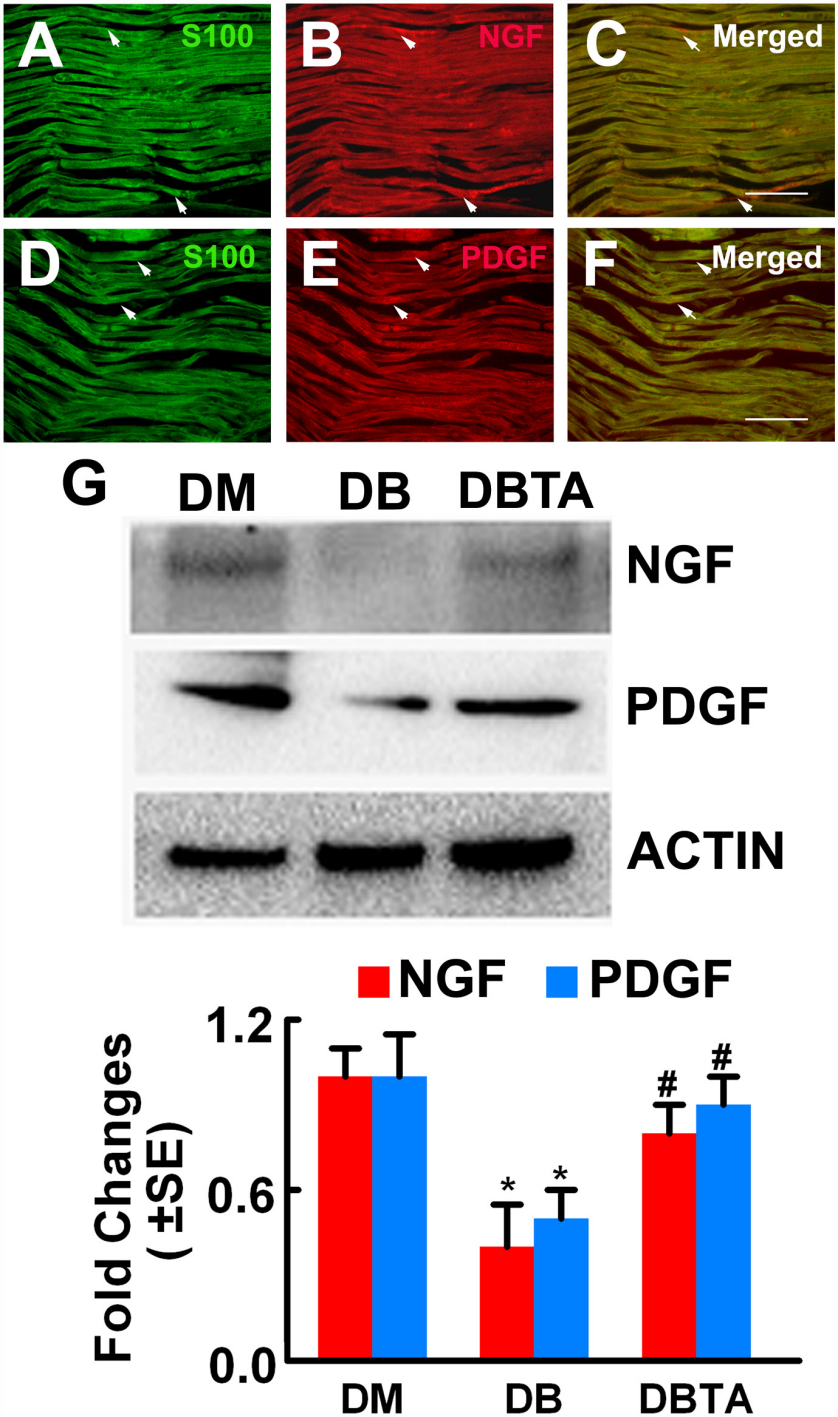
NGF and PDGF-C protein levels. Representative images of double immunofluorescent staining show that NGF and PDGF-C immunoreactivity (B, C, E, F, red, arrows) was co-localized to S100 positive Schwann cells (A, D, green, arrows). Western blot analysis (G) shows NGF and PDGF-C levels in the sciatic nerve tissue and β-actin was used as an internal control. *p<0.05 and #p<0.05 versus the non-diabetic mouse (DM) and the diabetic mouse treated with saline (DB), respectively. n = 6/group. Bar = 50μm. DBTA = diabetic mouse treated with tadalafil.

NGF and PDGF-C are neurotrophic factors, and have been shown to regulate neurovascular function. Using Western blot, we found that diabetic mice had a significant reduction of NGF and PDGF-C proteins in the sciatic nerve tissue compared to non-diabetic mice, whereas tadalafil treatment considerably increased NGF and PDGF-C compared to the saline treatment (Fig 5).

Moreover, double immunofluorescent staining showed that NGF and PDGF-C immunoreactivity was co-localized to S100 immunopositive Schwann cells in the sciatic nerve tissue (Fig 5). These data suggest that NGF and PDGF-C in Schwann cells may be involved in therapeutic effect of tadalafil on diabetic peripheral neuropathy.

## Discussion

The present study demonstrates that tadalafil is effective and safe for the treatment of diabetic neuropathy. Tadalafil significantly enhanced regional blood flow and functional vascular density in the sciatic nerve tissue as well as intraepidermal nerve density and sciatic nerve axonal remodeling, and concomitantly improved neurological functional recovery in diabetic mice with peripheral neuropathy.

We previously demonstrated that diabetes upregulated PDE5 expression in the sciatic nerve tissue, and suppression of PDE5 by sildenafil augmented vascular function and axonal remodel in the sciatic nerve tissue and subsequently improved neurological outcome in diabetic mice, indicating that sildenafil has a therapeutic effect on the diabetic neuropathy [6, 7]. The present study extends our previous work by showing that another PDE5 inhibitor, tadalafil, effectively improved functional recovery, concurrent with increase of neurovascular function without the reduction of blood glucose. Thus, tadalafil can achieve the comparable therapeutic effect on diabetic peripheral neuropathy as sildenafil does [6, 7].

Tadalafil is a long–acting PDE5 inhibitor and has a different chemical structure from sildenafil. In clinical trials, tadalafil is effective up to 36 hours after dosing, whereas the temporal effectiveness of sildenafil is 4 hours for the treatment of ED [25]. The current study shows that oral administration of tadalafil every 48 hours for 8 weeks improved functional recovery in diabetic mice, which is comparable to the therapeutic effect achieved by sildenafil with a daily dose for 8 weeks [7]. Our data demonstrate that tadalafil treatment provides additional therapeutic opportunities for the use of multiple PDE5 inhibitors in the treatment of peripheral neuropathy and possibly with less frequent administration than the short acting sildenafil. Thus, tadalafil could have potential clinical application for patients with long term diabetic peripheral neuropathy. However, additional experiments are warranted to investigate the effect of tadalafil and sildenafil on diabetic peripheral neuropathy by making a direct comparison between these two PDE5 inhibitors.

Although PDE5 is a modulator of the intracellular cGMP signaling pathway, PDE5 inhibitors may act on several downstream signaling effectors and subsequently regulate neurovascular function. NGF and PDGF-C are neurotrophic factors that not only promote vascular growth and maturation, but also directly regulate axonal remodeling by binding to their receptors, TrkA receptor and PDGF-α/β receptors, respectively [26–29]. In addition, NGF and PDGF mediate sildenafil neuroprotective effects in stroke and anti-proliferative effect on human pulmonary artery smooth muscle cells [30, 31]. The cGMP pathway contributes to NGF–mediated neurite outgrowth in DRG neurons derived from mice with sensory nerve defects and regulates PDGF-C-induced vascular smooth muscle and fibroblast cells migration [32, 33]. Recent studies have reported that NGF and PDGF-C appear to be important components of neurovascular interaction and crosstalk. Treatment with NGF and PDGF-C induced restoration of nerve function and revascularization, and concomitantly accelerated wound healing of the diabetic neuropathic foot [34, 35]. NGF also reversed peripheral neuropathic signs in animal models of diabetic neuropathy [36, 37]. The present study showed that diabetes reduced NGF and PDGF-C proteins in Schwann cells of the sciatic nerve tissue, whereas tadalafil treatment increased these two proteins, suggesting that NGF and PDGF-C may be involved in processes of diabetic peripheral neuropathy.

In contrast to a study by Varmus et al, who demonstrated that diabetic db/db mice at age of 12 weeks treated with tadalafil (1mg/kg, daily) for 4 weeks showed a significantly decrease in fasting plasma glucose levels and triglycerides [38]. Our results show that tadalafil treatment did not significantly change fasting plasma glucose and body weight in diabetic mice. The reasons for the discrepancy may be attributed to differences in animal age or treatment protocols. The present study demonstrates that treatment with tadalafil augments vascular function and axonal remodeling, which is associated with improved neurological function in diabetic mice with peripheral neuropathy. Thus, tadalafil may provide additional therapeutic opportunities for diabetic peripheral neuropathy.
